# Supplementary material for: A UMLS-based spell checker for natural language processing in vaccine safety
Source: BMC Med Inform Decis Mak. 2007 Feb 12;7:3. doi: 10.1186/1472-6947-7-3 (PMC1805499; doi:10.1186/1472-6947-7-3)
Supplement: Additional file 13 — RAP application directory. Sets up directory for RAP (RDF application for PHP) source code [file 1472-6947-7-3-S13.gz › rap/doc/phpdoc/elementindex_syntax.html]

xml version="1.0" encoding="iso-8859-1"?


## [syntax] element index

### Package indexes

- model
- dataset
- infModel
- resModel
- ontModel
- rdql
- utility

All elements
  

a
b
c
d
g
m
n
o
p
r
s
t
u
\_

\_

top

\_generateRDF
:   GRDDLParser::\_generateRDF() in GRDDLParser.php

\_is\_forbidden\_rdf\_property\_attribute
:   RdfParser::\_is\_forbidden\_rdf\_property\_attribute() in RdfParser.php

\_parse\_uri
:   RdfParser::\_parse\_uri() in RdfParser.php

\_report\_error
:   RdfParser::\_report\_error() in RdfParser.php

a

top

$anon
:   N3Serializer::$anon in N3Serializer.php

addNamespacePrefix
:   RdfSerializer::addNamespacePrefix() in RdfSerializer.php

    Adds a new prefix/namespace combination.

addNSPrefix
:   N3Serializer::addNSPrefix() in N3Serializer.php

    Adds a new namespace prefix to use.

b

top

$bNode
:   N3Parser::$bNode in N3Parser.php

c

top

configSortModel
:   RdfSerializer::configSortModel() in RdfSerializer.php

    Serializer congiguration: Sort Model Flag if the serializer should sort the model by subject before serializing.

configUseAttributes
:   RdfSerializer::configUseAttributes() in RdfSerializer.php

    Serializer congiguration: Use Attributes Flag if the serializer should serialize triples as XML attributes where possible.

configUseEntities
:   RdfSerializer::configUseEntities() in RdfSerializer.php

    Serializer congiguration: Use Entities Flag if the serializer should use entities for URIs.

configUseQnames
:   RdfSerializer::configUseQnames() in RdfSerializer.php

    Serializer congiguration: Use Qnames Flag if the serializer should use qualified names for RDF reserved words.

configUseXmlDeclaration
:   RdfSerializer::configUseXmlDeclaration() in RdfSerializer.php

    Serializer congiguration: Use XML Declaration Flag if the serializer should start documents with the xml declaration <?xml version="1.0" encoding="UTF-8" ?>.

d

top

$DAML\_NS
:   N3Parser::$DAML\_NS in N3Parser.php

$debug
:   NTripleSerializer::$debug in NTripleSerializer.php

$debug
:   N3Serializer::$debug in N3Serializer.php

$debug
:   N3Parser::$debug in N3Parser.php

$done
:   N3Serializer::$done in N3Serializer.php

g

top

generateModel
:   RdfParser::generateModel() in RdfParser.php

    Generates a new MemModel from a URI, a file or from memory.

generateModel
:   N3Parser::generateModel() in N3Parser.php

    Generate a new MemModel from an URI or file.

generateModel
:   GRDDLParser::generateModel() in GRDDLParser.php

    generates a MemModel and creates the DomDocument.

getPovs
:   N3Parser::getPovs() in N3Parser.php

    Gets a list of triples with same subject e.g. :Gunnar :firstname "Gunnar" ; :lastname "Grimnes.

GRDDLParser
:   GRDDLParser in GRDDLParser.php

    A GRDDLParser.

GRDDLParser.php
:   GRDDLParser.php in GRDDLParser.php

m

top

$model
:   NTripleSerializer::$model in NTripleSerializer.php

$model
:   RdfParser::$model in RdfParser.php

$model
:   N3Serializer::$model in N3Serializer.php

$m\_attributeStatements
:   RdfSerializer::$m\_attributeStatements in RdfSerializer.php

$m\_baseURI
:   RdfSerializer::$m\_baseURI in RdfSerializer.php

$m\_contentStatements
:   RdfSerializer::$m\_contentStatements in RdfSerializer.php

$m\_currentSubject
:   RdfSerializer::$m\_currentSubject in RdfSerializer.php

$m\_defaultNamespaces
:   RdfSerializer::$m\_defaultNamespaces in RdfSerializer.php

$m\_groupTypeStatement
:   RdfSerializer::$m\_groupTypeStatement in RdfSerializer.php

$m\_namespaces
:   RdfSerializer::$m\_namespaces in RdfSerializer.php

$m\_nextAutomaticPrefixIndex
:   RdfSerializer::$m\_nextAutomaticPrefixIndex in RdfSerializer.php

$m\_out
:   RdfSerializer::$m\_out in RdfSerializer.php

$m\_rdfAboutElementText
:   RdfSerializer::$m\_rdfAboutElementText in RdfSerializer.php

$m\_rdfIDElementText
:   RdfSerializer::$m\_rdfIDElementText in RdfSerializer.php

$m\_rdfResourceElementText
:   RdfSerializer::$m\_rdfResourceElementText in RdfSerializer.php

$m\_statements
:   RdfSerializer::$m\_statements in RdfSerializer.php

MAGIC\_STRING
:   MAGIC\_STRING in N3Serializer.php

n

top

N3Parser
:   N3Parser::N3Parser() in N3Parser.php

    Constructor

N3Parser
:   N3Parser in N3Parser.php

    PHP Notation3 Parser

N3Serializer
:   N3Serializer::N3Serializer() in N3Serializer.php

    Constructor

N3Serializer
:   N3Serializer in N3Serializer.php

    An abstract object.

NTripleSerializer
:   NTripleSerializer in NTripleSerializer.php

    PHP N-Triple Serializer

NTripleSerializer
:   NTripleSerializer::NTripleSerializer() in NTripleSerializer.php

    Constructor

N3Parser.php
:   N3Parser.php in N3Parser.php

N3Serializer.php
:   N3Serializer.php in N3Serializer.php

NTripleSerializer.php
:   NTripleSerializer.php in NTripleSerializer.php

o

top

$OWL\_NS
:   N3Parser::$OWL\_NS in N3Parser.php

p

top

$parsedNamespaces
:   N3Parser::$parsedNamespaces in N3Parser.php

$parseError
:   N3Parser::$parseError in N3Parser.php

$prefixes
:   N3Serializer::$prefixes in N3Serializer.php

parse
:   N3Parser::parse() in N3Parser.php

    This parses a N3 string and prints out the triples

parse2model
:   N3Parser::parse2model() in N3Parser.php

    This parses a N3 string and returns a memmodel

r

top

$RDF\_NS
:   N3Parser::$RDF\_NS in N3Parser.php

$rdf\_parser
:   RdfParser::$rdf\_parser in RdfParser.php

$rdf\_qnames
:   RdfSerializer::$rdf\_qnames in RdfSerializer.php

$rdf\_qname\_prefix
:   RdfSerializer::$rdf\_qname\_prefix in RdfSerializer.php

$res
:   N3Serializer::$res in N3Serializer.php

$res
:   NTripleSerializer::$res in NTripleSerializer.php

$resourcetext
:   N3Serializer::$resourcetext in N3Serializer.php

$resourcetext\_taken
:   N3Serializer::$resourcetext\_taken in N3Serializer.php

RdfParser
:   RdfParser in RdfParser.php

    An RDF paser.

RdfSerializer
:   RdfSerializer::RdfSerializer() in RdfSerializer.php

    Constructor

RdfSerializer
:   RdfSerializer in RdfSerializer.php

    An RDF seralizer.

RdfParser.php
:   RdfParser.php in RdfParser.php

RdfSerializer.php
:   RdfSerializer.php in RdfSerializer.php

s

top

$sort\_model
:   RdfSerializer::$sort\_model in RdfSerializer.php

saveAs
:   RdfSerializer::saveAs() in RdfSerializer.php

    Serializes a model and saves it into a file.

saveAs
:   NTripleSerializer::saveAs() in NTripleSerializer.php

    Serializes a model and saves it into a file.

saveAs
:   N3Serializer::saveAs() in N3Serializer.php

    Serializes a model and saves it into a file.

serialize
:   RdfSerializer::serialize() in RdfSerializer.php

    Serializes a model to RDF syntax.

serialize
:   N3Serializer::serialize() in N3Serializer.php

    Serializes a model to N3 syntax.

serialize
:   NTripleSerializer::serialize() in NTripleSerializer.php

    Serializes a model to N Triple syntax.

setFixBnodes
:   N3Parser::setFixBnodes() in N3Parser.php

    Sets, if BlankNode labels should be replaced by the generic label from the constants.php file

t

top

$Tokens
:   N3Parser::$Tokens in N3Parser.php

u

top

$use\_attributes
:   RdfSerializer::$use\_attributes in RdfSerializer.php

$use\_entities
:   RdfSerializer::$use\_entities in RdfSerializer.php

$use\_xml\_declaration
:   RdfSerializer::$use\_xml\_declaration in RdfSerializer.php

uparse
:   N3Parser::uparse() in N3Parser.php

    This parses a N3 string and calls func($subject, $predicate, $object) with each trioke

a
b
c
d
g
m
n
o
p
r
s
t
u
\_
